# Gut Microbiota and Neuroinflammation in Acute Liver Failure and Chronic Liver Disease

**DOI:** 10.3390/metabo13060772

**Published:** 2023-06-20

**Authors:** Lucia Giuli, Marta Maestri, Francesco Santopaolo, Maurizio Pompili, Francesca Romana Ponziani

**Affiliations:** 1 Internal Medicine and Gastroenterology-Hepatology Unit, Fondazione Policlinico Universitario Agostino Gemelli IRCCS, 00168 Rome, Italy; lucia.giuli92@gmail.com (L.G.); martamaestri4@gmail.com (M.M.); santopaolofrancesco@gmail.com (F.S.); maurizio.pompili@unicatt.it (M.P.); 2 Dipartimento di Medicina e Chirurgia Traslazionale, Università Cattolica del Sacro Cuore, 00168 Rome, Italy

**Keywords:** neuroinflammation, microglia, gut microbiota, gut–liver–brain axis, hepatic encephalopathy, ALF, chronic liver disease

## Abstract

Acute liver failure and chronic liver disease are associated with a wide spectrum of neurological changes, of which the best known is hepatic encephalopathy (HE). Historically, hyperammonemia, causing astrocyte swelling and cerebral oedema, was considered the main etiological factor in the pathogenesis of cerebral dysfunction in patients with acute and/or chronic liver disease. However, recent studies demonstrated a key role of neuroinflammation in the development of neurological complications in this setting. Neuroinflammation is characterized by activation of microglial cells and brain secretion of pro-inflammatory cytokines, such as tumor necrosis factor (TNF)-α, interleukin (IL)-1β, and IL-6, which alter neurotransmission, leading to cognitive and motor dysfunction. Changes in the gut microbiota resulting from liver disease play a crucial role in the pathogenesis of neuroinflammation. Dysbiosis and altered intestinal permeability, resulting in bacterial translocation and endotoxemia, are responsible for systemic inflammation, which can spread to brain tissue and trigger neuroinflammation. In addition, metabolites derived from the gut microbiota can act on the central nervous system and facilitate the development of neurological complications, exacerbating clinical manifestations. Thus, strategies aimed at modulating the gut microbiota may be effective therapeutic weapons. In this review, we summarize the current knowledge on the role of the gut–liver–brain axis in the pathogenesis of neurological dysfunction associated with liver disease, with a particular focus on neuroinflammation. In addition, we highlight emerging therapeutic approaches targeting the gut microbiota and inflammation in this clinical setting.

## 1. Introduction

Hepatic encephalopathy (HE) is one of the most important complications related to acute liver failure (ALF) and liver cirrhosis. It is characterized by a wide spectrum of neurological symptoms, ranging from subtle cognitive impairment to coma [1].

According to the type of underlying liver disorder, HE can be divided into type A, B, or C [2]. Type A is related to the development of ALF in patients without a previous history of liver disease, typically in the setting of acute viral infections, drug-induced liver injury, and vascular disorders. The presence of HE in patients with ALF correlates with high mortality rates, being characterized by cerebral oedema and intracranial hypertension, which may induce brain herniation. Type B HE is due to the presence of portosystemic shunts in the absence of underlying liver disease, while type C HE develops in patients with liver cirrhosis and is mainly characterized by impaired neurological function. It can present in two forms: minimal HE (MHE), which can be diagnosed only by psychometric tests, and overt HE, which is associated with symptoms of various degrees [2].

Whatever the type, HE is a predictor of poor prognosis, with not only a great impact on patients’ survival and quality of life, but also a heavy burden for caregivers [3].

Although HE pathogenic mechanisms are still not fully elucidated, ammonia was always considered the main causative factor [4]. However, it was shown that ammonia levels do not correlate with the severity of HE and that HE can also manifest in patients with normal ammonia levels, hinting at the presence of other contributing factors, such as systemic inflammation and oxidative stress [5,6]. Recently, several studies suggested a key role of neuroinflammation in this setting [7]. Indeed, systemic inflammation and hyperammonemia stimulate in concert neuroinflammation [8] through the activation of microglia and astrocytes with a propagation of the inflammatory response that correlates with the progression of HE [9]. In this context, gut microbiota gained increasing importance, as highlighted by the beneficial effect of gut-targeted therapies on the clinical manifestations of HE [10,11,12]. Gut microbiota derangement is associated with increased intestinal permeability, leading to bacterial translocation and endotoxemia, which are the main drivers of systemic inflammation, and therefore, potential triggers of neuroinflammation [13].

In this review we describe in detail the molecular mechanisms responsible for neuroinflammation. We analyze the inflammatory modifications at the brain level in both acute and chronic liver disease, also discussing the role of gut dysbiosis in this complex network.

The aim of this study is to emphasize the importance of the gut–liver–brain axis and gut microbiota influence on HE and neuroinflammation in order to better understand its pathogenesis, paving the way to the use of new therapeutic targets in the management of this severe liver disease-related complication. Indeed, beyond such standardized therapies as lactulose and rifaximin, other emerging gut-centered approaches, such as fecal microbiota transplantation (FMT), probiotics, postbiotics, and therapies targeting systemic inflammation including new derivatives of non-steroidal anti-inflammatory drugs (NSAIDS), are showing promising results and could play an important role in management of HE and neuroinflammation in the future.

## 2. Pathophysiology of Neuroinflammation

Neuroinflammation refers to the inflammatory response that develops within the central nervous system following several insults, such as infections, traumatic injury, or exposure to toxic metabolites [14]. Microglia and astrocytes, the main brain innate immune cells, drive this process by producing several pro-inflammatory cytokines, such as interleukin (IL)-1β, IL-6, and tumor necrosis factor (TNF)-α, chemokines, including C-C motif chemokine ligand 2 (CCL2), CCL5, and C-X-C motif chemokine ligand 1 CXCL1, secondary messengers, such as nitric oxide (NO) and prostaglandins, and reactive oxygen species [15]. Additionally, endothelial cells and peripheral immune cells act in promoting this inflammatory status [16].

Under normal conditions, the central nervous system (CNS) is protected from the entrance of potentially pathological agents into the cerebral circulation thanks to the blood–brain barrier (BBB), a highly selective structure made of endothelial cells and astrocytes [17]. The integrity of the BBB is guaranteed by tight junction (TJs) proteins such as occludin and claudin-5 [18]. Following injury and systemic inflammation, TJs undergo a dysregulation process that affects the integrity of the BBB, increasing the permeability of dangerous molecules that promote brain inflammation. Activated microglia and astrocytes in turn favor BBB dysfunction, exacerbating this process [19].

Microglia is made of CNS resident innate immune cells derived from myeloid lineage. They constitute about 10% of the total CNS cells [20]. Microglia play an active role in fundamental brain processes, such as neurogenesis, synaptic pruning and plasticity, and immune surveillance [21]. In physiological conditions, microglia are quiescent, but they actively monitor the surrounding parenchymal environment with their branching processes [22]. In response to stimulations such as pro-inflammatory cytokines or other pathological molecules, microglial cells become activated, undergoing a morphological transformation that allows them to reach the insulted site and produce inflammatory cytokines and chemokines to prevent CNS damage [23]. Thus, when chronically activated, microglia plays a key role in the paradoxical propagation of neuroinflammation, leading to neurodegeneration [24].

Astrocytes are the most abundant glial cells in the CNS, representing, with their processes, a critical component of the BBB together with endothelial cells [25]. Astrocytes also provide metabolic support to neurons, regulate cerebral blood flow, and modulate synapses formation and synaptic transmission, through the uptake and release of neurotransmitters [26,27]. Activated microglia releases IL-1α, TNFα, and complement 1q (C1q), and is responsible, together with peripheral inflammatory cytokines and signals, for astrocytes activation. In this way, a gliosis response occurs, which is characterized by the upregulation of glial fibrillary acid protein (GFAP) expression and gliotic scar formation [28,29]. Moreover, the inflammatory process activates the nuclear factor kappa B (NF-kB) signaling pathway that triggers astrocytes to release increasing amounts inflammatory cytokines, thus propagating neuroinflammation [30].

Neuroinflammation is normally part of a protective physiological process. However, its chronic and excessive activation triggers the development of brain damage with synaptic consequences, cell loss, and impaired neurogenesis [20] that, altogether, lead to manifestations related to nervous system dysfunctions, such as anxiety, depression, memory loss and cognitive impairment [13].

## 3. Gut–Liver Axis Contribution to Systemic Inflammation

The term gut microbiota refers to all the microorganisms, including bacteria, viruses, fungi, archaea, and protozoa that inhabit the human gut and live in a mutualistic and symbiotic relationship with the host [31].

More than 100 trillions of microorganisms form the gut microbiota, the composition of which varies along the gastrointestinal tract and is influenced by genetic and environmental factors, such as early life events (i.e., mode of delivery, breastfeeding), diet, lifestyle, and exposure to drugs [32]. Normally, the bacterial component of the gut microbiota consists of 90% anaerobic bacterial phyla, such as *Bacteroidetes* and *Firmicutes*, followed by *Proteobacteria* and *Actinobacteria* [33,34].

A growing body of evidence highlighted the importance of a balanced gut microbiota in maintaining host’s health given its role in several important functions for the organism [31,35,36,37]. Indeed, the gut microbiota is involved in the metabolism of undigested carbohydrates, producing short chain fatty acids (SCFAs) such as butyrate, propionate, and acetate, which not only are a source of energy for the organism and enterocytes [37,38], but also guarantee the integrity of the intestinal barrier and maintain intestinal motility [31]. Butyrate is mostly produced by *Firmicutes*, whereas acetate and propionate are mainly synthesized by *Bacteroidetes.* Butyrate intervenes in the maintenance of the gut barrier integrity by regulating tight junction proteins, such as claudin-1 and zonula occludens-1 [39]. In the liver, SCFAs control hepatic glucose and lipid homeostasis; in particular, propionate is involved in gluconeogenesis, while butyrate and acetate regulate lipogenesis. SCFAs, and especially butyrate, were shown to modulate the immune response, and consequently, liver inflammation [40].

Gut microbiota, and in particular, *Lactobacillus*, *Bifidobacterium*, and *Enterococcus*, convert primary bile acids derived from the liver into secondary bile acids, which exert antimicrobial effects and contribute to the homeostasis of the intestinal epithelial barrier and vascular barrier, through the interaction with the farnesoid X receptor (FXR) [41,42]. Moreover, the gut microbiota prevents colonization by pathogens, stimulates both the development of the innate and adaptive immune system, and synthesizes essential vitamins [43]. The disruption of gut microbiota composition, known as dysbiosis, is associated with the onset of various pathologies including liver, gastrointestinal, neurological, psychiatric, cardiovascular, and metabolic disorders [34].

In recent decades, increasing attention was paid to the close relationship between the gut microbiota and the liver. This strong bidirectional connection, known as the gut–liver–axis, is realized by the portal vein and the biliary tract; thus, gut-derived metabolites can reach the liver, which, in turn, releases bile acids as well as other mediators back into the intestine [44]. The intestinal barrier, composed of structural elements such as mucus layer, epithelial cells, vascular barrier, immune cells, and soluble mediators, plays a critical role in this interaction, limiting the systemic spread of toxins and pathogenic molecules [45]. To a certain extent, bacterial translocation, defined as the migration of bacteria and their products through the intestinal barrier into mesenteric lymph nodes (MLNs) or the portal venous system, is a physiological process and it is necessary for the modeling of the immune system [46]. Although through the portal vein the liver receives about 70% of its blood supply from the intestine, it is constantly exposed only to small amounts of bacteria and bacterial products that escaped from the surveillance of MLNs [47]. These products, in normal conditions, are eliminated by resident immune cells, such as Kupffer cells, dendritic cells, natural killer (NK) cells, and lymphocytes [48], so as to prevent their systemic spread, thus preserving a condition of immune tolerance [38,49].

Based on these premises, dysbiosis and the alteration of the intestinal barrier are correlated with the development and progression of liver disease. Many studies showed an important reduction in gut microbial diversity associated with liver disorders together with an increased relative abundance of pathogenic taxa (*Fusobacteria*, *Proteobacteria*, *Enterococcaceae*, and *Streptococacceae*) and depletion of the autochthonous ones, such as *Bacteroidetes*, *Ruminococcus*, *Roseburia*, *Veillonellaceae*, and *Lachnospiraceae* [50,51]. In cirrhotic patients, the ratio between beneficial bacteria (*Lachnospiraceae* + *Ruminococcaceae* + *Clostridium Cluster XIV* + *Veillonellaceae*) and potential pathogenic ones (*Enterobacteriaceae* + *Bacteroidaceae*), known as the cirrhosis/dysbiosis ratio (CDR), inversely correlates with model for end-stage liver disease (MELD) score and endotoxin [52]. These pathogens express active lipopolysaccharides (LPS) and promote hepatic and systemic inflammation when translocating [53].

Indeed, the loss of beneficial autologous taxa leads to a reduced production of SCFAs and conversion of primary into secondary bile acids that further exacerbate gut dysbiosis, alter the integrity of intestinal barrier and decrease gut motility, also favoring small intestinal bacterial overgrowth (SIBO) [12]. These alterations increase the rate of bacterial translocation and promote endotoxemia, with a huge amount of pathogen-associated molecular patterns (PAMPs) that reach the MLNs, with the consequent spread to the liver through the portal circulation [54,55,56].

Once in the liver, PAMPs interact with resident immune cells as Kupffer cells through Toll-like receptors (TLRs), which in turn promote myeloid differentiation primary response 88 (MyD88)-dependent and MyD88-independent molecular pathways bringing NF-kB activation, release of inflammatory cytokines as TNF-α, IL-1β, IL-6, IL-18, chemokines as CXCL1, CXCL2, CCL2, CCL5, CCL3, CCL4, NO, and reactive oxygen species [57].

These mechanisms cause a chronic inflammation of the liver, worsening liver impairment in a vicious circle, and contribute to systemic inflammation. All these alterations involving the composition and functions of the gut microbiota were strictly related to cirrhosis complications, including HE.

## 4. Role of the Gut Microbiota in Hepatic Encephalopathy and Neuroinflammation

A strong interplay was demonstrated between the gut microbiota and the central nervous system. This bidirectional network realizes the gut–brain–axis [13,58]. Different systems act together in this key channel, especially the enteric nervous system, the endocrine, and the immune system [59,60]. Indeed, the gut microbiota influences the function and development of the CNS by modulating signals via the vagus nerve, through the production of hormones and neurotransmitters, and the stimulation of the neuroimmune pathway by cytokines secretion [61]. On the other hand, the CNS uses these pathways to modulate intestinal secretory and immune functions, motility, and barrier permeability [62]. Alterations of the gut–brain–axis are correlated to the pathogenesis of several gastrointestinal, psychiatric, and neurological conditions such as irritable bowel syndrome, functional gastrointestinal disorders, major depression, anxiety, autism spectrum disorders, and neurodegenerative diseases [63,64,65]. In particular, it was found that gut-derived metabolites, such has Trimethylamine N-oxide (TMAO), synthesized by gut microbiota through enzymatic metabolism of choline, betaine, and carnitine, are strongly correlated with motor dysfunction and disease severity in patients with Parkinson’s disease [66,67].

In the setting of liver disease, HE is typically related to gut–liver–brain axis dysfunction. The pathogenesis of HE is still not fully clarified, although high brain ammonia levels were always considered a major etiological factor [68]. Ammonia is a by-product of nitrogen metabolism, principally derived from the metabolic activity of urease-producing bacteria in the gut and the deamination of glutamine by the enzyme glutaminase present in the enterocytes of the small intestine and the colon [4]. Other organs, such as muscles, brain, and kidney, participate to a lesser extent in ammonia metabolism [12]. In normal conditions, ammonia is transported to the liver through the portal vein, where it enters in the urea cycle and is converted into urea, which is subsequently excreted through the kidneys [69]. In case of liver dysfunction, ammonia metabolism is impaired, resulting in a significant increase in serum ammonia [70]. Ammonia has the capacity to cross the BBB; then, it is metabolized into glutamine by glutamine synthetase of the astrocytes. The excess of intracellular glutamine generates an osmotic gradient, leading to astrocytes swelling, increased activity of gamma-aminobutyric acid (GABA), brain oedema, and dysfunction [71] (Table 1). Hyperammonemia also exerts its deleterious role inducing neutrophil dysfunction, oxidative stress, and inflammation, and its effects are modulated by inflammatory mediators [72].

However, it was shown that ammonia levels do not correlate exactly with the severity of HE [73]. This indicates that other factors ranging from intestinal dysbiosis, systemic inflammation, and neuroinflammation intervene in the pathogenesis of HE [74]. Many studies confirmed the role of gut microbiota dysregulation in HE; therefore, most of the therapies used in its treatment act on microbiota modulation [70,74]. The over-abundance of ammonia in HE can be in part explained by an overgrowth of urease-producing bacteria, as demonstrated by the presence of a greater population of urease-producing *Proteobacteria* in patients with HE and poor cognition [75]. Bajaj et al. demonstrated an increased abundance of *Veillonellaceae*, poor cognition, endotoxemia, and inflammation (as indicated by serum levels of IL-6, TNF-, IL-2, and IL-13) in cirrhotic patients with HE compared to those without HE (Table 1). This study also showed a significant correlation between *Alcaligeneceae*, *Porphyromonadaceae*, *Enterobacteriaceae*, inflammatory parameters, and cognition [76]. Another study compared the sigmoid mucosal microbiome of cirrhotic patients with and without HE and controls [77]. Patients with HE had a worse MELD score and cognitive performance, together with higher IL-6 and endotoxin serum levels compared to patients without HE. Compared to cirrhotic patients, controls showed more autochthonous and less pathogenic taxa. Moreover, in correlations network analysis, genera overexpressed in HE as *Enterococcus*, *Megasphaera*, and *Burkholderia* were correlated to inflammation and poor cognition.

Ahluwalia et al. utilized magnetic resonance imaging (MRI) to characterize the association between cognition and specifical gut microbial families in patients with HE, demonstrating that *Enterobacteriaceae* were positively correlated with astrocytes changes typical of hyperammonemia at MRI. In addition, the presence of *Porphyromonadaceae* was only associated with neuronal damage in diffusion tensor imaging [78].

**Figure 1:**
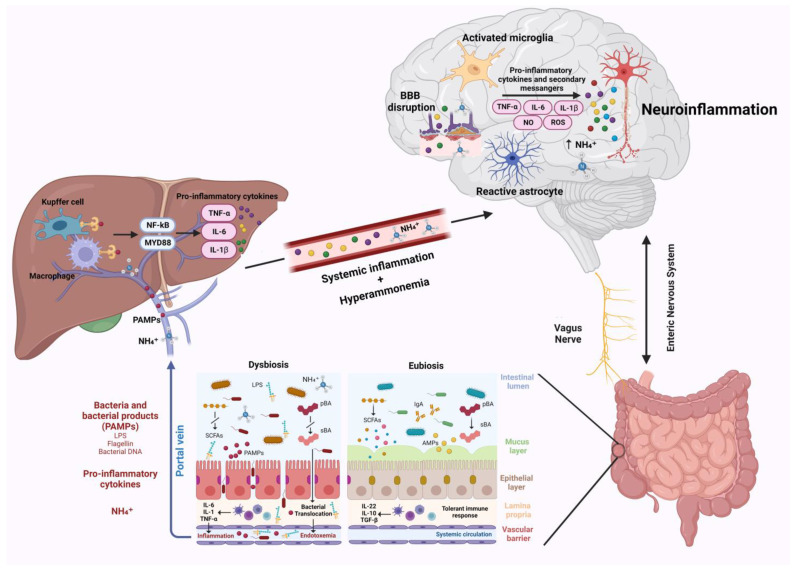
Gut–liver–brain axis and neuroinflammation. Gut dysbiosis and intestinal barrier impairment occurring during chronic liver disease lead to increased bacteria and their products/fragments that reach the liver through the portal vein. PAMPs interaction with TLR-4 on liver reticuloendothelial cells activates NF-kB and MyD88, leading to the release of pro-inflammatory cytokines, which trigger systemic inflammation. Systemic inflammation and hyperammonemia derived from impaired liver function cause BBB dysfunction, microglia, and astrocytes activation, which in turn promote neuroinflammation. Abbreviations: AMPs: antimicrobial peptides; BBB: blood brain barrier; IL: interleukin; LPS: lipopolysaccharide; MYD88: myeloid differentiation primary response 88; NF-kB: nuclear factor NF-kappa-B; NH_4+_: ammonium; NO: nitric oxide; PAMPs: pathogen associated molecular patterns; pBA: primary bile acid; ROS: reactive oxygen species; sBA: secondary bile acids; SCFAs: Short-chain fatty acids; TGF-β: transforming growth factor beta; and TNF-α: tumor necrosis factor α. Created with BioRender.com.

Neuroinflammation was recently suggested to represent another crucial factor in the pathogenesis of neurological impairment in liver disease [79]. Following liver dysfunction, hyperammonemia, circulating bile acids, and systemic inflammation are able to activate microglia, promoting neuroinflammation [7]. Gut microbiota, being one of the main actors in the development of systemic inflammation and in ammonia metabolism, plays a critical role in the pathogenesis of neuroinflammation (Figure 1) [13]. This relation establishes a connection between hepatic inflammation and neuroinflammation. Indeed, gut dysbiosis, SIBO, and intestinal barrier dysfunction lead to increased bacterial translocation and release in circulation of bacterial products, such as LPS, peptidoglycan, flagellin, and bacterial DNA [10,74]. These PAMPs interact with TLR-4 on the membrane of reticuloendothelial cells of the liver, such as Kupffer cells. This interaction in turn favors the activation of NF-kB and MyD88, triggering the release of inflammatory cytokines such as TNF-α, IL-6, and IL-1β by immune cells, leading to systemic inflammation [80,81,82]. This inflammatory process is responsible for blood–brain barrier dysfunction and neuroinflammation. As demonstrated in animal models, the aforementioned circulating inflammatory cytokines (TNF-α, IL-6, and IL-1β) may downregulate tight junction proteins claudin-5 and occludin of brain endothelial cells favoring BBB disruption [28,83]. This allows additional circulating harmful molecules, such as inflammatory cytokines, ions, and immune cells, to reach the brain, further affecting BBB permeability and promoting cerebral inflammation through the activation of microglia and astrocytes [19].

LPS, a component of the Gram-negative bacteria cell wall, represents one of the major contributors to systemic inflammation. Intravenous administration of LPS transiently caused systemic inflammatory responses with an increase in IL-6 and TNF-α serum levels [84] (Table 1). LPS, together with inflammatory cytokines and other factors (e.g., glutamate), promote microglial activation and the consequent release of inflammatory cytokines, leading to neuronal damage. Indeed, in the brain, LPS binds endothelial cell membrane receptors such as TLR-2, TLR-4, and CD14, leading to the release of secondary mediators responsible for oxidative stress and neuroinflammation [79].

TNF-α induces microglia to release CCL2, leading to the recruitment of monocytes in the brain and being responsible for neurological decline [85]. In an animal model of azoxymethane-induced ALF, the use of etanercept, a TNF-α neutralizing molecule, reduced both systemic and cerebral inflammation and prevented microglial activation [86].

A study by Bajaj et al. demonstrated the direct influence of the gut microbiota on neuroinflammation, comparing cirrhotic germ free (GF) mice with conventional cirrhotic mice. Although GF mice presented hyperammonemia unlike the GF non-cirrhotic counterparts, they did not show systemic inflammation or neuroinflammation. On the other hand, in conventional cirrhotic mice, which presented gut dysbiosis, higher levels of ammonia were found in association with systemic inflammation, neuroinflammation, and microglial activation. In particular, in these mice, there was a significant reduction in the abundance of intestinal autochthonous taxa, and a relative increase in *Staphylococcaceae*, *Lactobacillaceae*, and *Enterobacteriaceae*, which were closely correlated to systemic inflammation [87].

Another study further confirmed the close link between gut microbiota dysfunction and neuroinflammation in liver disorders. Similarly to conventional cirrhotic mice, GF mice colonized with stool from patients with cirrhosis showed enhanced neuroinflammation, microglial activation, and GABA signaling compared to GF mice that received stool from healthy donors. Moreover, GF mice colonized with stools from cirrhotic patients with HE who were previously treated with FMT presented a reduction in neuroinflammation [88].

Studies describing the role of gut microbiota in HE and neuroinflammation are shown in Table 1.

**Table 1:** Studies reporting on the role of gut microbiota in hepatic encephalopathy and neuroinflammation.

Study	Experimental Setting	Experimental Results	Clinical Results
Tofteng F. et al., 2006 [71]	Patients with fulminant hepatic failure (FHF).	↑ concentration of glutamine in the brain due to persistent arterial hyperammonemia. Brain concentration of glutamine correlated to ↑ intracranial pressure.	Persistent arterial hyperammonemia correlates with ↑ intracranial pressur and eventual cerebral herniation.
Shawcross D. L. et al., 2004 [72]	Patients with cirrhosis and clinical evidence of infection.	Hyperammonemia generated in response to the administration of aminoacids solution was similar prior to and after the resolution of inflammation.	↓ neuropsychological tests following induced hyperammonemia during the inflammatory status, but not after its resolution.
Shawcross D. L. et al., 2011 [73]	Cirrhotic patients with HE grade 3–4.	No difference in arterial ammonia/sodium/creatinine levels between patients with grades 3 and 4 HE.	Infection and systemic inflammation are associated with grades 3–4 HE and prognosis, not with serum ammonia.
Bajaj J. S. et al., 2012 [76]	Cirrhotic patients with and without HE.	↑ *Veillonellaceae*, endotoxemia, and inflammation (IL-6, TNF-α, IL-2, and IL-13) in cirrhotic patients with HE vs. without HE. ↑ *Enterobacteriaceae*, *Alcaligeneceae*, and *Fusobacteriaceae* and ↓ *Ruminococcaceae* and *Lachnospiraceae* compared with controls.	Gut microbiome is significantly different between healthy controls and cirrhotic patients, especially those with HE, and is associated with cognition impairment.
Bajaj J. S. et al., 2012 [77]	Cirrhotic patients with and without HE.	*↑ Enterococcus*, *Megasphaera*, and *Burkholderia* overexpressed in HE. ↑ IL-6 and endotoxin serum levels in HE.	↑ MELD score, poor cognition and inflammation are associated with HE.
Ahluwalia V. et al., 2016 [78]	Cirrhotic patients with and without HE.	In patients with HE, *Enterobacteriaceae* were positively correlated with astrocytes changes typical of hyperammonemia at MRI. ↑ systemic inflammation and ammonemia in HE. *Porphyromonadaceae* were associated with neuronal damage on diffusion tensor imaging.	↓ cognitive performance in patients with HE. Specific gut microbial taxa were related to neuronal and astrocytic changes associated with brain dysfunction in cirrhosis.
Labrenz F. et al., 2019 [84]	Healthy subjects.	↑ plasma IL-6 and TNF-α concentration, due to intravenous administration of LPS.	Systemic inflammation induced by LPS impaired functional connectivity in brain regions and networks implicated in emotion processing and regulation.
Seki E. et al., 2007 [82]	TLR4-chimeric mice.	The interaction of LPS with TLR-4 on the membrane of liver reticuloendothelial cells triggered the release of inflammatory cytokines such as TNF-α, IL-6, and IL-1β by immune cells.	This inflammatory process contributed to blood–brain barrier dysfunction and onset of neuroinflammation.
Kang D. J. et al., 2016 [87]	Cirrhotic GF and non-GF mice.	Hyperammonemia is not associated with systemic inflammation or neuroinflammation in cirrhotic GF mice. Relative ↑ *Staphylococcaceae*, *Lactobacillaceae* and *Enterobacteriaceae* and ↑ hyperammonemia in cirrhotic non-GF mice.	Gut dysbiosis was associated with systemic inflammation, neuroinflammation, and microglial activation.
Liu R. et al., 2020 [88]	GF mice.	↑ neuroinflammation, microglial activation, and GABA signalling in GF mice colonized with stools from patients with cirrhosis. ↓ neuroinflammation GF mice colonized with stools from cirrhotic patients with HE who were previously treated with FMT.	

↑: increased; ↓: decreased; FHF: fulminant hepatic failure; HE: hepatic encephalopathy; IL-6: interleukin-6; TNF-α: tumor necrosis factor-α; IL-2: interleukin-2; IL-13: interleukin-13; MELD: model for end-stage liver disease; MRI: magnetic resonance imaging; LPS: lipopolysaccharides; TLR-4: toll-like receptor 4; IL-1β: interleukin-1β; GF: germ free; GABA: gamma-amino-butyric-acid; and FMT: fecal microbiota transplantation.

## 5. Neuroinflammation in Acute Liver Failure and Chronic Liver Disease

Neuroinflammation appears to be implicated in the pathogenesis of both ALF and chronic liver disease. ALF is a life-threatening condition characterized by the development of HE and coagulative disorder in patients without a previous history of liver disease [89]. The mechanisms responsible for HE onset are not yet fully elucidated, although hyperammonemia and systemic inflammation, acting synergistically, appear to play an important role in its onset [90].

Recently, a growing body of evidence linked neuroinflammation to the development of CNS-related complications such as HE, brain oedema, brain herniation, and intracranial hypertension occurring in patients with ALF [91] (Table 2). Both microglia and astrocytes are involved in this process, producing local pro-inflammatory cytokines under the influence of systemic inflammatory signals deriving from the failing liver. Moreover, hyperammonemia causes astrocytes swelling, leading to brain edema [92]. Jiang et al., in 2006, demonstrated for the first time the presence of neuroinflammation in the brains of animals with induced ALF, showing the activation of microglia [93]. In another study involving sixteen patients with ALF, serum levels of pro-inflammatory cytokines, such as TNF-α, IL-1β, and IL-6, were elevated and significantly correlated with the severity of intracranial pressure [94]. In an animal model of ALF induced by hepatic devascularization, an increased expression of cluster of differentiation (CD)11b/c, which is a typical feature of microglia activation, was demonstrated. In addition, compared to controls, ALF rats have increased brain concentration of pro-inflammatory cytokines mRNA, and this correlates with HE progression and brain oedema, highlighting the contribution of neuroinflammation in ALF-related neurological complications [95].

Systemic inflammation during ALF also contributes to neuroinflammation. ALF induced by azoxymethane (AOM) in mice favors the release of hepatic transforming growth factor β 1 (TGFβ 1) into circulation, which binds TGFβ-receptor2 (TGFβR2) present on neurons, leading to the increase in CCL2 and decrease in CX3CL1 expression, which results in the activation of microglia. Indeed, neuroinflammatory responses were attenuated in mice receiving pharmacological inhibition of TGFβ1 or in TGFβR2 null mice [96].

Several studies also investigated the role of neuroinflammation in cognitive and motor alterations in MHE and overt HE in chronic liver disease [97,98,99] (Table 2). As in ALF, hyperammonemia and systemic inflammation seem to act synergically in the induction of neuroinflammation also in chronic liver disease.

Rats with induced chronic liver failure after portocaval shunt had increased brain levels of IL-6 and cyclooxygenase (COX) and inducible nitric oxide synthase (iNOS) activity, which are markers of neuroinflammation. These rats also presented a decreased function of glutamate-(NO)-cyclic guanosine monophosphate (cGMP), leading to cognitive impairment with a lower ability to learn Y-maze task. Treatment with the anti-inflammatory drug ibuprofen decreased neuroinflammation and restored rats’ cognitive ability through the normalization of NO-cGMP function, confirming the contribution of neuroinflammation in cognitive alterations of HE [100].

Rodrigo et al. also evaluated the role of neuroinflammation in a rat model of chronic liver injury and MHE obtained after bile duct ligation (BDL). Compared with controls, BDL rats showed activation of microglia and increased levels of IL-1β, prostaglandin E2, and iNOS activity in brain tissue samples, which were associated with cognitive and motor impairment. As previously reported, treatment with ibuprofen ameliorated cognitive and motor functions and reduced microglia activation [101]. A recent study also confirmed the presence of neuroinflammation with microglia morphological changes and astrocyte reactivity associated with BBB dysfunction in a rat BDL model of HE [102] (Table 2).

Evidence of neuroinflammation in patients with chronic liver disease and cognitive impairment is limited to few studies. Cagnin et al. demonstrated in five patients with cirrhosis and MHE who underwent positron emission tomography (PET) scans an increased binding of [11C]-PK11195 to peripheral benzodiazepine binding sites (PBBS) in the brain, confirming microglia activation. The highest increase in [11C](*R*)-PK11195 binding was seen in the patient with the worst cognitive impairment [103]. Another analysis of post-mortem human brain tissue from cirrhotic patients with and without HE and non-cirrhotic controls found increased levels of ionized calcium-binding adaptor molecule-1 (Iba-1), another marker of microglia activation, in the brain of cirrhotic patients with HE but not in patients without HE. However, unlike previous studies, microglia activation was not associated with differential expression of pro-inflammatory cytokines in the cerebral cortex [104] (Table 2).

Taken together, these findings suggest neuroinflammation involvement in cognitive and motor dysfunction in HE associated with chronic liver disease, although more studies are needed to reinforce this hypothesis.

**Table 2:** Studies reporting the characteristics of microglia activation and neuroinflammation both in acute liver failure and chronic liver disease.

Study	Model Studied	Experimental Results	Clinical Results
Wright G. et al., 2007 [94]	Patients with ALF (n. 16).	↑ brain proinflammatory cytokines (TNF-α, IL-6 and IL-1β).	Progression of HE correlated with the degree of proinflammatory cytokines expression in the brain.
Cagnin A. et al., 2006 [103]	Patients with MHE and biopsy proven cirrhosis subjected to PET (n. 5).	↑ expression of PBBS by glial cells.	Severity of cognitive impairment correlated with the expression of PBBS.
Zemtsova I. et al., 2011 [104]	Post mortem brain tissue from patients with cirrhosis with (n. 8) and without HE and non-cirrhotic controls (n. 8).	↑ Iba-1 in the cerebral cortex from patients with cirrhosis and HE.	HE in patients with ↑ Iba-1.
Jiang W. et al., 2009 [95]	ALF rats at coma stages of HE.	↑ TNF-α, IL-6 and IL-1β in the brain and cerebrospinal fluid.	Proinflammatory cytokines in the brain correlated with the onset of brain edema and the progression of HE.
McMillin M. et al., 2019 [96]	Mice model of ALF.	↑ TGFβ 1 which binds TGFβR2 on neurons leading to ↑ CCL2 and ↓ CX3CL1.	Neurological decline (attenuated by TGFβ 1 inhibition).
Zemtsova I. et al., 2011 [104]	Rats with acute ammonium acetate intoxication.	↑ Iba-1 in the cerebral cortex from acutely ammonia-intoxicated rats.	
McMillin M. et al., 2014 [99]	Mouse model of azoxymethane induced ALF.	↑ CCL2, ↑ microglia activation.	CCL2 correlates with microglia activation and neurological decline.
Cauli M. et al., 2007 [100]	Rat model of chronic liver failure.	↑ brain IL-6, COX, and iNOS. ↓ NO-cGMP functions.	Cognitive impairment.
Rodrigo R. et al., 2010 [101]	Rat model of chronic liver injury after BDL.	↑ IL-1b. ↑ prostaglandin E2 and iNOS.	Cognitive and motor impairment.
Claeys W. et al., 2022 [102]	Mouse model of HE in chronic liver disease after BDL.	Hyperammonemia. Brain ammonia overload (with ↑ glutamine, ↓ taurine, and choline). Microglial morphological changes. BBB disruption.	Motor dysfunction.
Dhanda S. et al., 2018 [98]	Rat model of chronic liver injury after BDL.	↑ TNF-α, IL-6 and MCP-1 ↓ GFAP and Iba-1.	Cognitive impairment.

↑: increased; ↓: decreased; ALF: acute liver failure; TNF-α: tumor necrosis factor α; IL-6: interleukin-6; IL-1β: interleukin-1β; HE: hepatic encephalopathy; MHE: minimal hepatic encephalopathy; PET: positron emission tomography; PBBS: peripheral benzodiazepine binding sites; Iba-1: calcium-binding adaptor molecule-1; TGFβ 1: transforming growth factor β 1; TGFβR2: TGFβ-receptor2; CCL2: C-C motif chemokine ligand 2, CX3CL1: C-X3-C motif chemokine ligand 3; COX: cyclooxygenase; iNOS: inducible nitric oxide synthase; NO: nitric oxide; cGMP: cyclic guanosine monophosphate; BDL: bile duct ligation; MCP-1: monocyte chemoattractant protein-1; and GFAP: glial fibrillary acidic protein.

## 6. Intestinal Microbiota Modulation as Treatment Strategy and Emerging Therapies

Several compounds that exert modulating effects on the gut microbial community, but also drugs with anti-inflammatory properties, were proven to beneficially affect neuroinflammation in liver disease.

### 6.1. Rifaximin

Rifaximin is an eubiotic compound currently approved for the treatment of overt HE [2,105]. Several studies looked at how rifaximin can help the nervous system recover from neuroinflammation.

Mangas-Losada et al. administered rifaximin 1200 mg/day for six months to 22 cirrhotic patients with MHE. No significant changes in liver function, hemoglobin, or ammonia serum level were found, while immunological alterations showed a remarkable improvement in responder patients. In particular, pro-inflammatory CD14++CD16+ monocytes decreased in favor of anti-inflammatory CD14++CD16− monocytes; auto-reactive CD4+CD28− T-lymphocytes also decreased, while non-reactive CD4+CD28+ T-lymphocytes increased with the disappearance of CD69, a marker of early activation. Th22 CD4+ subsets and follicular Th diminished, as well as many pro-inflammatory cytokines, and levels of immunoglobulins normalized. Conversely, non-responders showed only a reduction in IL-6, CCL20, and T lymphocytes differentiation to Th22, and did not present increased expression of CD69 before treatment [106].

Another study conducted in rat models of MHE with mild liver damage similar to non-alcoholic fatty liver disease (NAFLD) [107] showed hippocampal neuroinflammation with consequent spatial learning and memory impairment. Increase in CCL2 levels in the hippocampus could be an early event that promotes microglia activation and monocytes infiltration, with their conversion in macrophages. Microglia activation leads to TNFα increase in the hippocampus, which generates the downregulation of NR1 and NR2 subunits of N-methyl-D-aspartate (NMDA) receptors, responsible for the impairment in spatial learning and memory. Daily administration of rifaximin 20 mg/kg counteracts this process, normalizing NMDA expression and improving cognitive functions. However, some alterations, such as infiltration of CD4+ lymphocytes, astrocytes activation, IL-1 increase, and enhanced membrane expression of the GluA2 subunit of α-amino-3-hydroxy-5-methyl-4-isoxazolepropionic acid (AMPA) receptors, were not reversed. Rats also showed T lymphocytes and macrophages infiltration in cerebellum, which is associated with motor incoordination. Early rifaximin treatment 20 mg/kg/day prevented the increase in TNFα, CCL20, and CX3CL1 in both plasma and cerebellum, IL-17 and IL-15 in plasma, and CCL2 in cerebellum, restoring motor coordination [108].

Other evidence in mice showed that rifaximin reduces neuroinflammation and cognitive impairment through microbiota modulation and promotion of the gut barrier integrity [109] (Table 3). Furthermore, rifaximin favors the growth of gut bacteria associated with production of SCFAs [110], which are able to cross the BBB and exert anti-inflammatory properties [111]. Indeed, in rats with chronic unpredictable mild stress (CUMS)-induced depression-like behaviors, administration of rifaximin 150 mg/kg/day for 4 weeks increased butyrate brain concentration and improved behavioral alteration. It was proven in vitro that butyrate induced a functional transformation of microglia towards an anti-inflammatory phenotype, reducing the release of TNFα and IL1β and phagocytic activity, also favoring the secretion of IL-10 [110]. Some studies report only a slight modification in the gut microbiota composition after rifaximin treatment in cirrhotic patients with HE, but a significant change in serum metabolomics due to gut microbiota end products, including SCFAs, in addition to a remarkable improvement in endotoxemia and hyperammonemia [112,113].

Rifaximin thus appears to be an effective drug for restoring the neurocognitive function in HE. Its direct action on microglia opens up new horizons for its use as a frontline treatment for MHE or HE; its modulating action on the microbiota also appears to be crucial from the perspective of preventing and counteracting HE.

### 6.2. Lactulose

Lactulose is a nonabsorbable disaccharide approved for the treatment, prevention, and secondary prophylaxis of overt HE [114]. Some evidence showed effectiveness also in MHE and covert HE [2].

Both systemic inflammation and hyperammonemia, which lead to lactate accumulation in the brain, are responsible for microglial activation and neuroinflammation and contribute to HE [101,115]. Studies in both rat models and cirrhotic patients with MHE demonstrate that lactulose administration lowers serum endotoxins and pro-inflammatory cytokines such as TNFα, IL-2, IL-6, IL-13, and IL-18 [76,116,117]. Through its cathartic action and the acidification of the intestinal environment, lactulose also reduces ammonia levels in the blood. Indeed, gut bacteria metabolize lactulose producing SCFAs, such as lactic and acetic acid, which lower colonic pH. An acidic environment decreases the content of urease-producing bacteria and favors the production of non-absorbable ammonium (NH4+), which cannot pass the gut barrier [118]. Of note, a randomized multicentre controlled trial, evaluating the effects of lactulose 30–60 mL/day in 98 cirrhotic patients with MHE, showed a different gut microbial profile between lactulose responders and non-responders. Particularly, lower abundance of potential pathogenic *Proteobacteria*, in addition to reduced metabolism of aminoacids and carbohydrates and lower serum ammonia levels, were found in responders [119] (Table 3).

A recent pre-clinical study in rats showed that lactulose is effective in alleviating methamphetamine-induced neurotoxicity, suppressing oxidative stress and neuroinflammation trough the up-regulation of the antioxidant system nuclear factor erythroid 2-relatted factor-2/heme oxygenase-1 (Nrf2/HO-1) directly in the striatum [120].

These data confirm the importance of using lactulose in HE at all stages and for its prevention. In fact, lactulose is an effective agent that reduces inflammation directly at the neuronal level and also reduces systemic inflammation and hyperammoniemia through the gut microbiota modulation.

### 6.3. Non-Steroidal Anti-Inflammatory Drugs (NSAIDs)

A recent study using rat models of HE with hyperammonemia reported a beneficial effect of the NSAID ibuprofen. A significant improvement in spatial memory and anxiety was registered after treatment with ibuprofen; the combination of ibuprofen and the antioxidant 1,8-cineol also increased the superoxide dismutase activity and significantly reduced oxidative stress [121]. Another pre-clinical study in rat models of HE reported a complete reversal of hypokinesia due to increased extracellular glutamate in substantia nigra pars reticulata (SNr) in rats with portacaval shunts (PCS) treated with ibuprofen 30 mg/kg. At the molecular level, this therapy normalized the amount of glutamate transporters GLT-1 and of excitatory amino acid carrier 1 (EAAC-1) and decreased by 53% extracellular glutamate in SNr of PCS rats [122].

Despite these positive results, NSAID therapy is burdened by unacceptable toxicities, such as renal damage and gastropathy in cirrhotic patients [123,124]. Therefore, Augusti et al. tested SB239063, an inhibitor of the piridinyl imidazol family, in PCS rats as a potential new drug, aiming to reduce neuroinflammation, in the absence of the adverse effects of NSAIDs. SB239063 reduced microglia activation through the inhibition of the p30 mitogen-activated protein kinase (MAPK), usually increased in the brains of PCS rats [125]; moreover, brain (prostaglandin E2, cyclooxygenase activity, iNOS, IL-1β, and TNFα) and blood (prostaglandin E2 and TNFα) inflammatory markers decreased, although ammonia and glutamine levels were not affected. In addition, SB239063 completely restored learning ability, coordination, and motor activity without altering creatinine or sodium levels.

These studies open promising scenarios toward new drugs that can effectively treat HE. Further investigations are needed to elucidate the mechanisms of action underlying NSAID effectiveness in this setting. Other studies analyzing molecules capable of inhibiting microglia activation, in the absence of the adverse effects of ibuprofen, are already underway.

### 6.4. Fecal Microbiota Transplantation

FMT, through its ability to modulate gut microbiota, can potentially reverse all the consequences of gut dysbiosis, such as increased gut barrier permeability, bacterial translocation, and systemic inflammation. Several animal models suggested a beneficial effect of FMT on neuroinflammation.

In a rat model of HE induced by the administration of CCl4, FMT was able to improve cognitive functions and HE, improved gut barrier permeability and significantly decreased ammonia serum levels and the expression of TLR4 and TLR9, two important receptors involved in the inflammatory response. Overall, these effects led to a strong reduction in pro-inflammatory cytokines such as IL-1β, IL-6, and TNF-α, pointing out how FMT could be useful in modulating systemic inflammation and, consequently, neuroinflammation [126] (Table 3). Indeed, GF mice colonized with stools from patients with liver cirrhosis and HE who were previously treated with FMT presented a reduction in neuroinflammation [88].

In a randomized clinical trial, 20 patients with cirrhosis and recurrent HE were randomly assigned to receive either 15 capsules of FMT from a single donor enriched in *Lachnospiraceae* and *Ruminococcaceae* or placebo. The FMT group showed increased duodenal microbiota diversity with higher abundance of *Ruminococcaceae* and *Bifidobacteriacceae* associated with a reduction in *Streptococcaceae* and *Veillonellaceae*. These changes in the gut microbiota composition were accompanied by the increase in E-cadherin and defensin alpha 5 and the concomitant reduction in pro-inflammatory cytokines, such as IL-6 and lipopolysaccharide binding protein (LBP) [127].

Although the current evidence outlines FMT potential benefits for HE and neuroinflammation, larger clinical studies are needed to standardize its use and eventually validate FMT treatment in patients with HE.

### 6.5. Probiotics, Prebiotics and Postbiotics

Several studies report the gut microbiota modulatory properties of probiotics, thus they are supposed to help in counteracting the mechanisms of neuroinflammation and HE in patients with liver disease. Probiotics reduce the overgrowth of pathogenic bacteria, maintain the integrity of tight junction proteins strengthening the gut barrier, and decrease intestinal bacteria translocation, with consequent reduction in endotoxemia and systemic inflammation [128,129,130,131,132,133].

In addition, *Lactobacillus* has the ability to inhibit gut urease-producing bacteria and to acidify intestinal environment with the consequent reduction in serum ammonia levels [134].

A randomized controlled trial involving 120 cirrhotic patients who recovered from an episode of HE proved the superiority of VSL#3, a group of eight probiotics, over placebo in improving Child Turcotte Pugh (CTP) and MELD scores and lowering the rate of HE recurrence and hospitalization [131]. However, the effectiveness of probiotics compared to lactulose is uncertain [135].

A recent Cochrane meta-analysis of 21 trials including 1420 participants with HE of any grade showed that probiotics, mainly VSL#3, may confer advantages in quality of life, development of overt HE, and ammonia concentration, when compared to placebo or no intervention. However, probiotics did not show any statistically significant advantage in terms of mortality when compared to placebo or lactulose. Nevertheless, the quality of the available evidence is very low, and further investigations are needed [136].

A recent clinical trial involving 125 patients with viral or cryptogenic chronic cirrhosis, with grade 1 or 2 HE and hyperammonemia, showed that synbiotics, which are the combination of probiotics plus prebiotics, can be a valuable alternative to lactulose [137]. Patients were randomized to receive either *Bifidobacterium* and fructo-oligosaccharides or lactulose for 60 days. Both treatments proved effective in reducing serum ammonia and improving psychometric test scores and cognitive function. No adverse effects were reported in the *Bifidobacterium* and fructo-oligosaccharides group, while in the lactulose group, there were a few cases of gastrointestinal complaints such as diarrhea, cramps or flatulence.

According to a meta-analysis by Shukla et al., lactulose is more effective than prebiotics, probiotics, and synbiotics in the treatment of MHE, but synbiotics and probiotics also result in improved MHE with the benefit of improved tolerability [138].

Another study conducted in 69 cirrhotic patients with overt HE, showed how synbiotics in combination with branched chain amino acids were effective in improving cognition, compared with placebo, although there was no significant change in ammonia serum levels [139].

Currently, the evidence in literature regarding the benefits and efficacy of all these agents in treating HE and reducing neuroinflammation is still too weak to allow their introduction into clinical practice for this purpose.

**Table 3:** Studies reporting on the effects of therapies targeting the gut microbiota on neuroinflammation.

Study	Agent Studied	Experimental Setting	Experimental Results	Clinical Results
Meng D. et al., 2022 [109]	Rifaximin.	Mice with CRD.	Rifaximin modulated gut microbiota, improved intestinal barrier integrity and ↓ inflammatory response.	↓ cognitive impairment induced by CRD.
Li H. et al., 2021 [110]	Rifaximin.	Rats exposed to CUMS.	Rifaximin ↑ the relative abundance of *Ruminococcaceae* and *Lachnospiraceae*, ↑ anti-inflammatory factors released by microglia.	↓ depressive-like behaviour induced by CUMS.
Liu R. et al., 2022 [88]	FMT.	GF rats.	↑ neuroinflammation, activation of GABAergic and neuronal activation in GF rats receiving FMT from cirrhotic patients. ↓ neuroinflammation in GF rats colonized with stools from cirrhosis patients with HE who were previously treated with FMT.	
Wang W. W. et al., 2017 [119]	FMT.	Rat model of HE induced with CCl4.	↓ TLR4 and TLR9 in the liver. ↓ circulating IL-1β, IL-6 and TNF-α. Restoration of tight junction proteins in the intestinal tissue.	↑ behaviour, spatial learning capability and HE grade.
Kaji K. et al., 2017 [112]	Rifaximin.	Patients with decompensated cirrhosis (n. 20).	↓ relative abundance of *Veillonella* and *Streptococcus* ↓ endotoxins.	↑ cognition.
Bajaj J. S. et al., 2013 [113]	Rifaximin.	Cirrhotic patients with MHE (n. 20).	↓ endotoxemia. Modest ↓ in *Veillonellaceae* and ↑ in *Eubacteriaceae.*	↑ cognition.
Bajaj J. S. et al., 2019 [127]	FMT.	Cirrhotic patients with recurrent HE and MELD < 17 receiving standard of care therapy (n. 20).	↑ gut microbiota diversity with ↑ abundance of *Ruminococcaceae* and *Bifidobacteriacceae*, ↓ *Streptococcaceae* and *Veillonellaceae*. ↑ E-cadherin and defensin alpha 5. ↓ pro-inflammatory cytokines.	↑ cognition.
Wang J. Y. et al., 2019 [119]	Lactulose.	Cirrhotic patients with MHE (n. 98).	↓ abundance of potentially pathogenic *Proteobacteria*, ↓ metabolism of amino acids and carbohydrates and serum ammonia levels in responders.	↑ MHE recovery rate.

↑: increased; ↓: decreased; CRD: circadian rhythm disruption; CUMS: chronic unpredictable mild stress; FMT: fecal microbiota transplant; GF: germ free; GABA: gamma-amino-butyric-acid; HE: hepatic encephalopathy; CCl4: carbon tetrachloride; TLR-4: toll-like receptor 4; TLR-9: toll-like receptor 9; IL-1β: interleukin-1β; IL-6: interleukin-6; TNF-α: tumor necrosis factor-α; MHE: minimal hepatic encephalopathy; and MELD: model for end-stage liver disease.

### 6.6. Challenges of Proposed Treatments

Rifaximin and lactulose are effective, low-cost drugs characterized by a good tolerability and safety profile [140,141,142]; however, some adverse effects, such as bloating, may reduce quality of life and decrease the adherence to therapy [141]. In addition, in cirrhotic patients, circulating levels of rifaximin, which has usually a negligible absorption, may rise due to increased intestinal permeability, with the risk of altering the safety profile of the drug [143].

Several studies demonstrate the efficacy of FMT as a new treatment of HE. However, there is still no standardization about the route of administration, dosage, or the ideal bacterial consortium to be adopted for the transplant. This is made more difficult by the fact that each donor has a peculiar microbiome, which is complex to analyze [144,145]. Up to date, FMT appears to be a safe treatment, although risks for the potential bacterial dissemination in the bloodstream were reported [146].

Various studies show that probiotics and synbiotics have a higher tolerability profile than lactulose, but the efficacy is not superior. Additionally for probiotics, there is still no standardization of either the ideal components or the amounts to be administered, making the clinical studies conducted so far very heterogeneous.

Lastly, NSAIDs seem to be effective in counteracting mechanisms of neuroinflammation; however, the adverse effects involving the kidney and the gastrointestinal tract make these drugs unsuitable for cirrhotic patients; new molecules similar to NSAIDs, but with a better safety profile, are being developed in the pre-clinical stage and represent hope for future application in clinical practice.

## 7. Conclusions

Brain inflammation contributes to the neurological changes observed in patients with HE. Neuroinflammation finds its roots in the dysfunctional response of innate immune cells in the brain, namely astrocytes and microglia. Systemic inflammation has an important role in the pathogenesis of such activation, as it damages the BBB, making it more permeable to factors that activate microglia and astrocytes. The activation of such cells results in the release of host cytokines and proinflammatory mediators, which in turn damage the BBB, fueling a vicious cycle. Nevertheless, systemic inflammation is a prolonged insult, the effects of which in the brain are amplified by hyperammonemia, another hallmark of liver disease. Indeed, ammonia can cross the BBB, leading to astrocyte swelling, neutrophil dysfunction, oxidative distress and alteration of GABAergic transmission.

Several studies in recent years showed that gut microbiota modulation and restoration of intestinal eubiosis are key elements in counteracting HE in both early and advanced stages. Indeed, a “healthy” gut microbiota promotes the integrity of the intestinal barrier, reduces bacterial translocation, circulating LPS, and systemic inflammation, ultimately decreasing brain injury and microglia activation; nevertheless, it harbors fewer urease-producing bacteria and consequently lower circulating levels of ammonia.

Therefore, two roads are clearly emerging in the treatment of HE: the use of agents that modulate the gut microbiota, and the use of anti-inflammatory drugs that act directly and indirectly on brain inflammatory pathways.

This may become a cue for the development of new drugs that can turn off neuroinflammation at different levels, even by acting at an early stage on microglia activation. A growing interest is also in understanding the differences between those patients who respond to treatment and those who do not, which is important especially in identifying a personalized therapeutic strategy. Finally, a major focus is on the effect that rifaximin and lactulose have on gut microbiota metabolomics rather than on its composition. In the future, molecules interfering with SCFAs metabolism, or rather SCFAs themselves, deserve further investigation for the treatment of HE. In this regard, as the most innovative therapy with an extraordinary ability to modulate gut microbiota, FMT, gained great attention in recent years. However, more evidence is needed to support its use in clinical practice to modulate neuroinflammation.

Some limitations of the studies conducted so far can be explained by the uncertainty of the mechanisms underlying neuroinflammation and the lack of standardized diagnostic criteria and outcome measures for neuroinflammation in liver disease. In addition, the various studies reported have limited numbers of patients and short treatment periods; nevertheless, several factors, such as etiology, disease severity, comorbidities, and interaction with the external environment, influence both neuroinflammation and the gut microbiota. Gut microbiota characterization may vary depending on the technique used, and there is still no standardization about the administration of the various agents. All these concerns can make it difficult to compare results across studies and to determine the optimal treatment approach.

Despite these limitations, there is evidence that these new therapeutic strategies may be promising, and it is worth addressing the challenge of shedding more light on their safety and efficacy for a better management of HE in a personalized approach.

In conclusion, neuroinflammation appears to be a promising and blooming area of study for the treatment and prevention of HE. The currently available therapeutic strategies appear to be partially effective in modulating neuroinflammation, so it is desirable to identify new effective weapons that are also easily applicable in clinical practice.

## Abbreviations

HE	hepatic encephalopathy
TNF	tumor necrosis factor
IL	interleukin
ALF	acute liver failure
MHE	minimal hepatic encephalopathy
NSAIDS	non-steroidal anti-inflammatory drugs
CCL	C-C motif chemokine ligand
CXCL	C-X-C motif chemokine ligand
NO	nitric oxide
CNS	central nervous system
BBB	blood–brain barrier
TJs	tight-junctions
C1q	complement 1q
GFAP	glial fibrillary acid protein
NF-kB	nuclear factor kappa B
SCFAs	short chain fatty acids
FXR	farnesoid X receptor
NK	natural killer
SIBO	small intestinal bacterial overgrowth
GABA	gamma-aminobutyric acid
MELD	model for end-stage liver disease
MRI	magnetic resonance imaging
LPS	lipopolysaccharides
PAMPs	pathogen-associated molecular patterns
TLR	toll-like receptor
MyD88	myeloid differentiation primary response 88
GF	germ free
FMT	fecal microbiota transplantation
CD	cluster of differentiation
mRNA	messengerRNA
AOM	azoxymethane
TGF	transforming growth factor
TGFβR2	transforming growth factor β-receptor2
COX	cyclooxygenase
iNOS	inducible nitric oxide synthase
cGMP	glutamate-(NO)-cyclic guanosine monophosphate
BDL	bile duct ligation
PET	positron emission tomography
PBBS	peripheral benzodiazepine binding sites
CD	cluster of differentiation
NAFLD	non-alcoholic fatty liver disease
NMDA	N-methyl-D-aspartate
AMPA	α-amino-3-hydroxy-5-methyl-4-isoxazolepropionic acid
CUMS	chronic unpredictable mild stress
NH4+	ammonium
Nrf2/HO-1	nuclear factor erythroid 2-relatted factor-2/heme oxygenase-1
SNr	substantia nigra pars reticulata
PCS	portacaval shunts
GLT	glutamate transporters
MAPK	p30 mitogen-activated protein kinase
CTP	Child Turcotte Pugh
BCAAs	branched chain amino acids
